# Modulation of Working Memory Using Transcranial Electrical Stimulation: A Direct Comparison Between TACS and TDCS

**DOI:** 10.3389/fnins.2018.00761

**Published:** 2018-10-23

**Authors:** Franziska Röhner, Carolin Breitling, Katharina S. Rufener, Hans-Jochen Heinze, Hermann Hinrichs, Kerstin Krauel, Catherine M. Sweeney-Reed

**Affiliations:** ^1^Neurocybernetics and Rehabilitation, Department of Neurology and Stereotactic Neurosurgery, Otto von Guericke University Magdeburg, Magdeburg, Germany; ^2^Department of Child and Adolescent Psychiatry and Psychotherapy, Otto von Guericke University Magdeburg, Magdeburg, Germany; ^3^Department of Neurology, Otto von Guericke University Magdeburg, Magdeburg, Germany; ^4^Center for Behavioral Brain Sciences (CBBS), Magdeburg, Germany; ^5^Department of Behavioral Neurology, Leibniz Institute for Neurobiology, Magdeburg, Germany; ^6^German Center for Neurodegenerative Diseases, Magdeburg, Germany

**Keywords:** brain stimulation, TACS, TDCS, working memory, n-back

## Abstract

Transcranial electrical stimulation (TES) has been considered a promising tool for improving working memory (WM) performance. Recent studies have demonstrated modulation of networks underpinning WM processing through application of transcranial alternating current (TACS) as well as direct current (TDCS) stimulation. Differences between study designs have limited direct comparison of the efficacy of these approaches, however. Here we directly compared the effects of theta TACS (6 Hz) and anodal TDCS on WM, applying TACS to the frontal-parietal loop and TDCS to the dorsolateral prefrontal cortex (DLPFC). WM was evaluated using a visual 2-back WM task. A within-subject, crossover design was applied (*N* = 30) in three separate sessions. TACS, TDCS, and sham stimulation were administered in a counterbalanced order, and the WM task was performed before, during, and after stimulation. Neither reaction times for hits (RT-hit) nor accuracy differed according to stimulation type with this study design. A marked practice effect was noted, however, with improvement in RT-hit irrespective of stimulation type, which peaked at the end of the second session. Pre-stimulation RT-hits in session three returned to the level observed pre-stimulation in session two, irrespective of stimulation type. The participants who received sham stimulation in session one and had therefore improved their performance due to practice alone, had thus reached a plateau by session two, enabling us to pool RT-hits from sessions two and three for these participants. The pooling allowed implementation of a within-subject crossover study design, with a direct comparison of the effects of TACS and TDCS in a subgroup of participants (*N* = 10), each of whom received both stimulation types, in a counterbalanced order, with pre-stimulation performance the same for both sessions. TACS resulted in a greater improvement in RT-hits than TDCS (*F*(2,18) = 4.31 *p* = 0.03). Our findings suggest that future work optimizing the application of TACS has the potential to facilitate WM performance.

## Introduction

The use of transcranial electrical stimulation (TES) to manipulate cognitive function is a rapidly growing area of investigation, with applications ranging from modification of learning processes in healthy participants to treatment of neurological and psychiatric disease (Coffman et al., 2014; Prehn-Kristensen et al., 2014; Tortella et al., 2015; Breitling et al., 2016; Dagan et al., 2018). Given the low cost and ease of use, it has the potential for wide application, both in enhancing cognitive processes in healthy participants as well as in improving cognitive function in disease. Two widely used TES-techniques are alternating (TACS) and direct current (TDCS). Common practice is to choose one of these methods and compare behavioral outcome measures in response to active versus sham or anodal versus cathodal stimulation. The mechanisms of action of TACS and TDCS are indeed likely to differ substantially, so that their further development will require separate approaches. Our aim was to make a direct comparison between the effects of TACS and TDCS in healthy participants, matching the parameters of the methods as closely as possible, given their fundamental differences, in a within-subject, crossover design. We investigated working memory (WM) function for two reasons. Firstly, WM is crucial to daily life, so that its enhancement has potentially wide application. Second, both TACS and TDCS have been shown to have an impact on WM. While TDCS has thus far received more attention (review: Polanía et al., 2018), TACS offers the potential advantage of providing a direct modulation of ongoing brain oscillatory activity known to underpin the relevant cognitive processing (Polanía et al., 2012; Herrmann et al., 2013; Jausovec and Jausovec, 2014).

Transcranial direct current is considered to alter the neuronal firing threshold through up- or down-regulation of neuronal resting membrane potentials, with anodal TDCS increasing excitability of the underlying cortex (Creutzfeldt et al., 1962; Bindman et al., 1964). Anodal TDCS has been shown to enhance WM performance (Fregni et al., 2005; Ohn et al., 2008; Andrews et al., 2011; Zaehle et al., 2011), but the results are mixed, and include both negative findings as well as small changes in WM performance (Brunoni et al., 2011; Hill et al., 2016; Mancuso et al., 2016). It has been suggested that the lack of reproducibility of some positive findings is the result of wide variation in study parameters (Kim et al., 2014; López-Alonso et al., 2014; Wiethoff et al., 2014; Polanía et al., 2018).

The sinusoidal current applied using TACS means that the net membrane potential is unaltered. The mechanism of action of TACS is therefore likely to be based on entrainment of ongoing cortical activity rather than local alterations in cortical excitability (Antal and Herrmann, 2016). A potential advantage of TACS is that the stimulation frequency is chosen on a physiological basis, with the aim of modulating known, task-relevant physiological processes. The choice of stimulation frequency is dependent on the specific task under investigation, as demonstrated in a series of studies comparing the effects of TACS using different stimulation frequencies. Beta-TACS specifically resulted in enhanced short-term memory capacity (Feurra et al., 2016) and increased voluntary risky decision making (Yaple et al., 2017), while fluid intelligence was enhanced using gamma-TACS (Santarnecchi et al., 2013). Neurophysiological evidence suggests that WM, which involves the processing, storage, and manipulation of incoming verbal and visual information (Baddeley and Hitch, 1974), is driven by frontal-parietal networks oscillating in the theta (4–8 Hz) frequency range (Sarnthein et al., 1998; Prabhakaran et al., 2000; Palva et al., 2010; Sweeney-Reed et al., 2012, 2014). Direct manipulation of this activity using TACS is therefore a hypothesis-driven intervention, and indeed applying TACS in the theta frequency range has yielded promising results, including improvement in WM capacity, reaction times (RT), and accuracy (Polanía et al., 2012; Jausovec and Jausovec, 2014; Jausovec et al., 2014; Meiron and Lavidor, 2014; Violante et al., 2017). We therefore applied TACS at 6 Hz, in the middle of the theta range (Polanía et al., 2012; Violante et al., 2017).

While both TACS and TDCS have yielded improvements in WM performance, the variation in study designs, parameters, and participant groups limits a direct comparison between these approaches based on the literature. Here we directly compared the effects of TACS and TDCS on WM performance, in which the experimental conditions were as similar as possible. We applied TACS in the theta-frequency range (6 Hz) to the frontal-parietal network, stimulating left F3 and P3, in accordance with a study design in which TACS positively influenced WM (Polanía et al., 2012), and targeted the left dorsolateral prefrontal cortex (DLPFC) with TDCS over F3, also based on previous improved WM performance (Zaehle et al., 2011).

We compared WM processing using a visual 2-back task for three reasons. Firstly, the paradigm is well-established in the literature. The common application of the n-back test in TES studies of WM has importantly allowed comparison between studies on review and meta-analysis (Brunoni and Vanderhasselt, 2014; Hill et al., 2016; Mancuso et al., 2016). Second, it is deemed to activate both DLPFC and parietal cortex (Owen et al., 2005) and should thus engage the stimulated brain regions. Third, studies applying theta-TACS (Jausovec et al., 2014; Meiron and Lavidor, 2014; Violante et al., 2017) and studies using anodal TDCS (Fregni et al., 2005; Zaehle et al., 2011) have reported enhancement of WM performance using an n-back task. Although the WM task differed in the study by (Polanía et al., 2012), it similarly involved retention in WM of items from a series.

## Materials and Methods

### Participants

Thirty healthy adults aged 20 – 32 years [M (*SD*) = 26.2 (± 3.0); N (female) = 30 (15)] were recruited via the study participant register of the Leibniz Institute for Neurobiology, Magdeburg (LIN) and public notice. All participants were right-handed, had received no TES prior to this study, and had no history of neurological or psychiatric disorder or of drug abuse. We adhered to well-established safety guidelines (Nitsche et al., 2008). All participants underwent the KAI short-form intelligence test (Lehrl et al., 1992) to ensure an IQ over 85 [M(*SD*) = 120.8 ( ± 11.5)]. There was no difference in age, gender, or IQ between the groups receiving stimulation type in a particular order. To address the potential influence of circadian rhythms on performance, each participant received stimulation and testing at the same time of day in all three sessions. The appointment times did not differ significantly between *stimulation types* [one-way repeated measures ANOVA: *F*(2,58) = 2.33 *p* = 0.11]. We concluded that the impact of circadian rhythm on performance was balanced over the three stimulation types. The compensation was 8 Euro per hour. At the beginning of the study, participants received verbal and written information about the procedure, including potential side-effects, and all provided written consent. Our study was approved by the ethics committee of the Medical Faculty of the Otto-von-Guericke University, Magdeburg, following the ethical standards of the Helsinki Declaration.

### Study Design and Task

**FIGURE 1:**
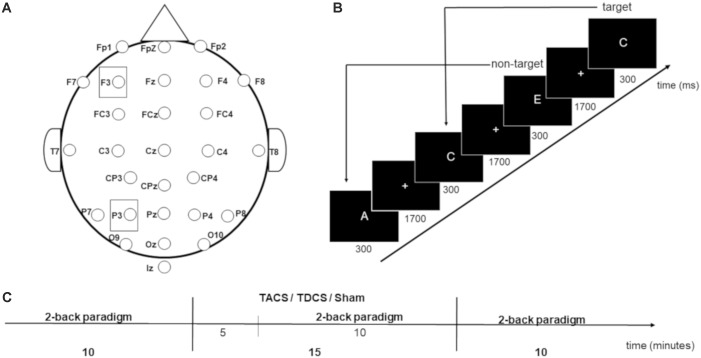
**(A)** Electrode placement. **(B)** 2-back working memory task. **(C)** Procedure during one session.

Each participant underwent three sessions, in which TACS, anodal TDCS, or sham stimulation was applied. Because 7 days between sessions is considered adequate to eliminate potential carry-over effects of stimulation (Andrews et al., 2011), each participant received three appointments on the same weekday for three consecutive weeks. The sequence of stimulation types was counterbalanced pseudo-randomly at the beginning of the study, and gender was balanced between the groups receiving stimulation in each particular order. The study was single-blinded (for blinding conditions, see section “Transcranial Electrical Stimulation”). During each session, participants performed a visual 2-back letter task three times for 10 min (pre-, during, and post-stimulation). The stimulation duration was 15 min. Each letter was presented for 300 ms, followed by a fixation cross (1300 ms) (Figure 1). The participants were instructed to respond as quickly and accurately as possible as to whether the letter shown matched the letter shown two letters previously. Responses were given via button press using the left or right index finger (counter-balanced across participants). In 10 min, 300 letters (A–E) were presented, 75 of which were targets. The task was performed using Presentation Software (Version 18.2 Build 02.18.16) and shown on a 24 inch screen. Letters were 1.5 cm tall (white) on a black background. A distance of 0.85 m resulted in a visual angle of 1.001°.

### Transcranial Electrical Stimulation

Transcranial electrical stimulation was applied using a battery driven DC-stimulator (NeuroConn, DC-Stimulator Plus serial 2049 Version 4.3.00.17). Rubber electrodes (5 cm × 7 cm) were placed under an EEG cap with electrode locations marked according to the international 10–20 system to achieve correct stimulation electrode placement. Electrodes were covered with saline soaked sponges (0.9% saline solution) to improve conduction and avoid skin irritation (Dundas et al., 2007). Stimulation intensity was 1 mA for all stimulation types. To achieve participant blinding to the stimulation condition, the same electrode placement was used for all sessions: the active electrodes were positioned over electrode sites F3 and P3 (Figure 1), and the reference electrode was placed in an extracephalic location, at the top of the left shoulder, to avoid shunting of the current and potential confounding effects of cathodal stimulation at another cephalic site (Creutzfeldt et al., 1962; Bindman et al., 1964; Stagg and Nitsche, 2011). TACS was applied by splitting the stimulation electrodes in two, leading to 500 μA intensity under F3 and P3 (current density 14.28 μA/cm^2^). The frequency was set to 6 Hz and with 0° phase difference (in-phase), in accordance with the protocol applied by Polanía et al. (2012). When TDCS was applied, only the electrode placed at F3 was connected to the stimulation device to provide anodal stimulation (1 mA intensity, with density of 28.57 μA/cm^2^ under F3), following the protocol applied by Zaehle et al. (2011). The device was placed behind the participant so that it was impossible to see which electrode cable was plugged into the stimulator. One minute of TDCS was applied in the sham condition to ensure participant blinding to stimulation condition, because skin side-effects to TES are generally only noted by participants in the first minute of stimulation. To minimize skin sensation for all three stimulation types, current intensity was ramped up at the beginning and ramped down at the end for 15 s. The impedance under each electrode was maintained under 20 kΩ.

### Statistics

For each item, the participant’s first response was taken. To evaluate WM performance, RTs were analyzed for hits (RT-hit), which is the most commonly reported measure with which to evaluate performance in the n-back task (Zaehle et al., 2011; Violante et al., 2017). We also calculated d-prime (d′) for each 2-back letter task. The d′ is a well-established measure to quantify WM performance and assess whether it has changed over time (Haatveit et al., 2010). The d′ value is based on subtracting the false-alarm-rate from the hit-rate, thus providing a reliable measure of the participant’s ability to discriminate between items. Hits indicates correct responses, while false alarm items are the letters to which the participant incorrectly answered that the letter had been shown two items previously. We chose to evaluate d′ rather than a percentage score for hits, because a participant who simply responded that a letter had been seen two items before every time would achieve a 100% hit rate, which would not reflect an ability to discriminate between the items.

Because d′ and RTs measure different aspects of performance in a 2-back task, we considered combining these measures, using the inverse efficiency score (IES) (Townsend and Ashby, 1978; Bruyer and Brysbaert, 2011). Certain conditions must be fulfilled for the IES to be applied. The accuracy should exceed 90% (Bruyer and Brysbaert, 2011; Isella et al., 2015), and there should be a high, linear correlation between the percentage of errors and the RTs to correct responses. The percentage of errors did not correlate with the RTs for correct responses for any of the stimulation types (Pearson’s correlation: TACS: *r* = -0.29, *p* = 0.112; TDCS: *r* = -0.17, *p* = 0.37; sham: *r* = -0.28, *p* = 0.14). Moreover, the accuracy rates did not exceed 90% (TACS: 85%; TDCS = 85%; sham = 84%). The conditions for the application of the IES were thus not fulfilled (Townsend and Ashby, 1978; Bruyer and Brysbaert, 2011; Isella et al., 2015).

Pre-stimulation performance for all sessions, quantified using RT-hits and d′, was compared between stimulation types using one-way ANOVAs with the within-subject factor of *stimulation type* (TACS, TDCS, sham stimulation). The pre-stimulation baseline did not differ according to *stimulation type* [RT-hits: *F* (2,58) = 0.12, *p* = 0.88; d′: *F*(2,58) = 0.24, *p* = 0.79].

Firstly, a two-way repeated measures ANOVA was conducted for RT-hits, with the within-subject factors of *stimulation type* (TACS vs. TDCS vs. sham) and *assessment time* (pre- vs. during vs. post-stimulation) (*N* = 30; within-subject crossover design). Second, a one-way repeated measures ANOVA with the factor *time* (nine levels: pre-, during, and post-stimulation each of the three sessions) was calculated to examine changes in RT-hits over the course of the whole study to reveal potential learning effects (Breitling et al., 2016). These ANOVAs were also calculated for accuracy (d′).

A within-subject, crossover design was used. However, a pronounced practice effect was found in the first session, irrespective of stimulation type, that masked any performance changes related to the applied stimulation condition (i.e., TACS, TDCS, and sham). Because the practice-related improvement reached a plateau after the second stimulation session, we performed a subgroup analysis including only the participants that had received sham stimulation in the first session. This approach allowed analysis of the differential effect of TACS and TDCS on task-performance without being confounded by learning effects. A two-way repeated measures ANOVA was then conducted for RT-hits with the within-subject factors of *stimulation type* (TACS vs. TDCS) and *assessment time* (pre- vs. during vs. post-stimulation). A separate ANOVA was calculated for accuracy. Mauchly′s test of sphericity was applied, and violations were corrected using the Greenhouse-Geisser method.

Outliers were identified by applying a RT and d′ threshold two standard deviations above or below the mean value across all participants. All initial ANOVAs were calculated with and without outliers, and the outcomes were the same. We considered exclusion of the outliers in further analyses, but in the absence of an independent measure by which they could be defined as differing from a normal study population, we chose to retain them for all subsequent analyses. Our primary aim was indeed to compare the effects of TACS with TDCS for application in a healthy population, and retaining the full data set provides a more robust evaluation than selecting cases.

Statistical analysis was performed using IBM SPSS (Version 24) software and in-house Matlab scripts (Mathworks, Version R2015b).

## Results

### Reaction Times

**FIGURE 2:**
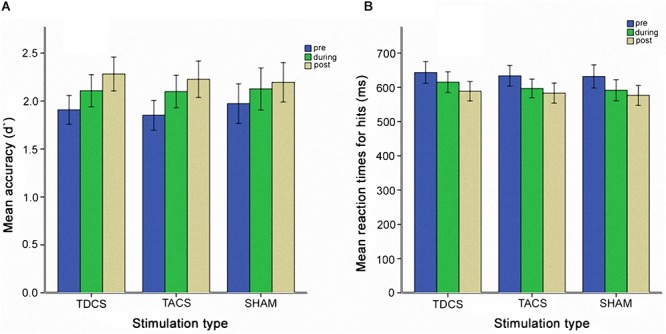
Behavioral performance measured before, during, and after stimulation for each stimulation type. **(A)** Mean accuracy reflected in d′ values. **(B)** Mean reaction times for hits. Error bars represent one standard error of the mean.

The two-way repeated measures ANOVA did not reveal a significant interaction between *stimulation type* and *assessment time* [*F*(4,74) = 0.53, *p* = 0.71] (Figure 2B). A simple main effect of assessment time was identified, however [*F*(1.54,29.24) = 12.91, *p* < 0.005, Greenhouse-Geisser corrected: 𝜀 = 0.77], indicating a significant effect of practice, independent of stimulation type.

**FIGURE 3:**
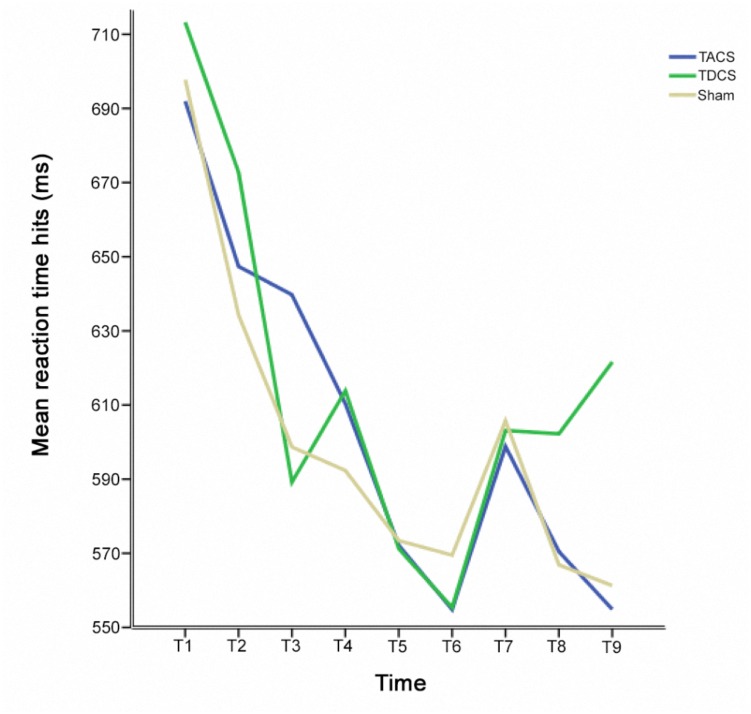
Mean reaction times for hits before, during, and after stimulation at each assessment time for each stimulation type.

**FIGURE 4:**
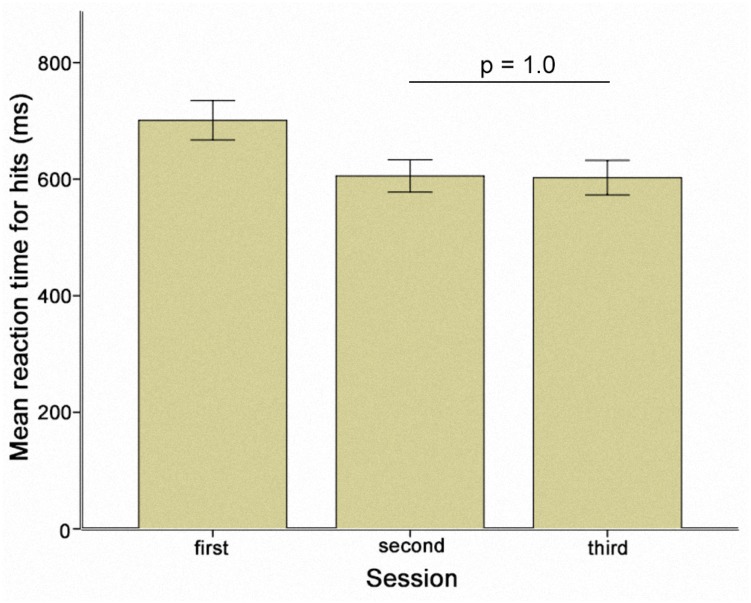
Mean reaction times for hits prior to stimulation separately demonstrated for the three sessions. Error bars represent one standard error of the mean.

The change in RT-hits over all experimental sessions was examined by calculating a one-way repeated measures ANOVA [*F*(2.67,778.17) = 14.11; *p* < 0.005; Greenhouse-Geisser corrected: 𝜀 = 0.34]. Maximal improvement was identified at the end of the second session (Figure 3). The pre-stimulation performance level in session three then returned to the same level as the pre-stimulation performance in session two (*p* = 1.0) (Figure 4). As a result, sessions two and three could be regarded as providing a measure of individual performance in the absence of a practice effect. We therefore performed a subgroup analysis based on the performance of participants who received sham stimulation in the first session (*N* = 10). Selecting this subgroup meant that the participants had reached the limits of the benefits of practice, and further effects resulting from stimulation type (TACS vs. TDCS) could be directly compared. Applying a two-way repeated measures ANOVA revealed a two-way interaction between stimulation and time [*F*(2,18) = 4.31; *p* = 0.03].

*Post hoc* tests were performed using one-way repeated measures ANOVAs. To investigate a simple main effect of stimulation type at each time point (before, during, and after stimulation), we conducted three one-way repeated measures ANOVAs with the within-subject factor *stimulation typ*e (TDCS, TACS) for the time points pre [*F*(1,9) = 0.007; *p* = 0.94], during [*F*(1,9) = 0.55; *p* = 0.48] and after stimulation [*F*(1,9) = 5.04; *p* = 0.051].

To evaluate the simple main effect of assessment time (before, during, and after stimulation) for each stimulation type separately, two one-way repeated measure ANOVAs with the within-subject factor *time (*pre, during, and post) were calculated. TDCS resulted in no such main effect of *time* [*F*(2,18) = 0.55; *p* = 0.059], whereas TACS did show a main effect of *time F*(2,18) = 6.29; *p* = 0.008].

**FIGURE 5:**
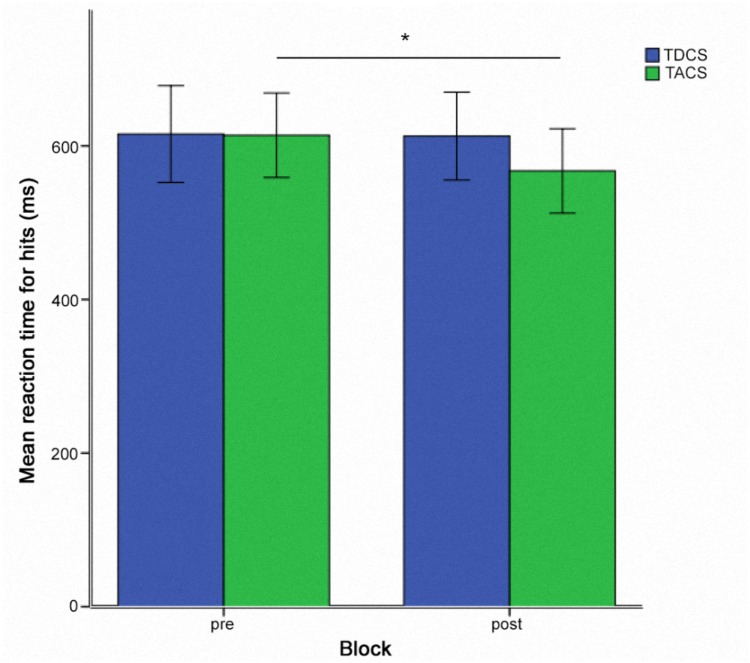
Reaction times for hits for sessions two and three pooled together for TACS and TDCS, in a within-subject crossover design (*N* = 10). ^∗^ = *p* < 0.05. Error bars represent one standard error of the mean.

Pairwise *post hoc t*-tests comparing all time points in the TACS condition revealed a significant difference between pre- and post-stimulation RTs [*t*(9) = 3.39; *p* = 0.008] (Figure 5), whereas the differences between pre- and during stimulation [*t*(9) = 2.05; *p* = 0.07] and during and post-stimulation RTs [*t*(9) = 1.50; *p* = 0.17] were not significant.

**FIGURE 6:**
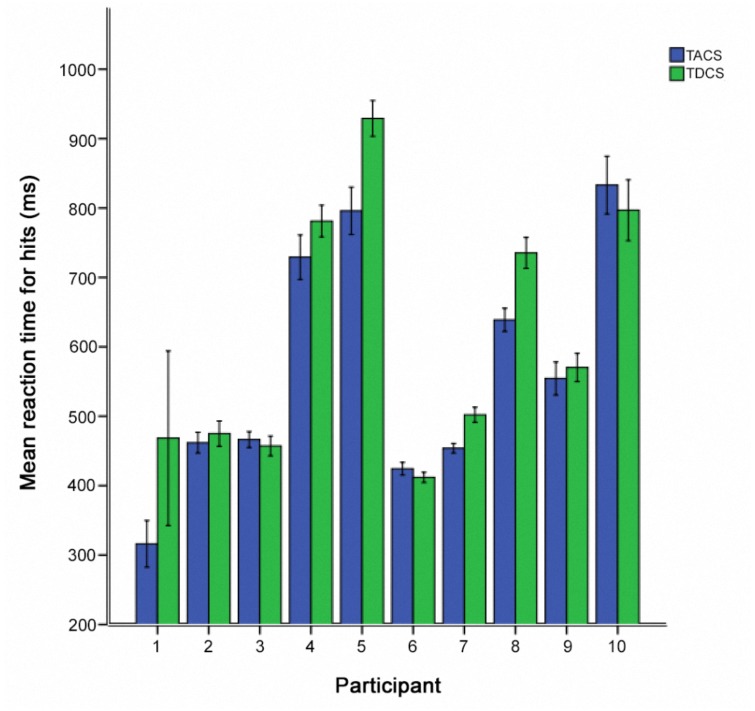
Individual post-stimulation reaction times for hits, comparing stimulation type for participants receiving sham-stimulation in the first session (*N* = 10). Error bars represent one standard error of the mean.

We additionally examined the intra- and inter-individual differences in RT-hits following TACS and TDCS in the participants who received sham stimulation in the first session. In 7 of 10 participants, a greater RT improvement was seen following TACS than TDCS (Figure 6).

### Accuracy

We analyzed changes in the d′ analogously to the RTs. A two-way repeated measures ANOVA showed a main effect only of *time* [*F*(2,58) = 24.71, *p* < 0.005] but no interaction of *time* and *stimulation type* [*F*(2.98,86.54) = 0.56, *p* = 0.64, Greenhouse-Geisser corrected: 𝜀 = 0.75] (Figure 2A). During a session, accuracy improved, but there was no association with stimulation type. A practice effect was observed using a one-way repeated measures ANOVA [*F*(2.17,62,87) = 15.31, *p* < 0.005, Greenhouse-Geisser corrected: 𝜀 = 0.27]. *Post hoc* tests showed significant improvement only during the first session. Based on the result of the ANOVA, we also analyzed the second and third sessions pooled for participants receiving sham stimulation in the first session (*N* = 10), analogously to the RT analysis. No interaction between *stimulation group* and *time* was observed [*F*(2,18) = 0.93, *p* = 0.41], and no main effect of *stimulation group* or *time* was seen.

We note that participants were not able to distinguish between stimulation type, indicating successful blinding.

## Discussion

Despite rapidly growing interest in applying TES to modulate cognitive function, reports in the literature are mixed, and further work is required to identify the most promising lines for future development. We investigated whether there is a difference in modulation of WM performance using theta TACS compared with anodal TDCS. We evaluated the impact on WM using the visual 2-back test, because behavioral measures of performance using this paradigm are well-established, and TES has been reported to influence these measures. However, the extent of the improvement in RT-hits over time under all three stimulation conditions (TACS, TDCS, and sham stimulation) outweighed any effects of TES, rendering the original within-subject crossover study design unsuitable for our purposes. Detailed exploration of the changes in RTs over the course of the entire study, however, revealed the evolution of differential effects of TACS and TDCS when we controlled for learning effects. Performing the task three times in the first session, in the absence of stimulation (i.e., taking the group that started with the sham condition), meant that the participants had learned to carry out the task, with an improvement in performance resulting from practice independently of stimulation. The pre-stimulation RT-hits across all participants in session three returned to the level observed pre-stimulation in session two (Figure 4). This return to baseline performance levels permitted pooling of the TACS and TDCS findings for these two sessions, which allowed restoration of a within-subject design, in which the order of TACS and TDCS was counterbalanced across the participants. This approach provided the opportunity to analyze stimulation effects independently of the practice effect. The offline effect of TACS stimulation (comparing pre- and post-stimulation RTs) revealed a significant improvement in performance, which was not observed in the TDCS condition.

We also identified a practice effect with respect to accuracy over the course of the entire study. Accuracy increased steadily until the end of session three, but only improvements made during the first session were significant. We note that our finding that only RTs were influenced by TES is in accord with previous reports (Zaehle et al., 2011; Polanía et al., 2012). A ceiling effect is plausible, however, given evidence that a 2-back WM task involves a low WM load. On the other hand, our participants reported finding the 2-back task demanding. While we did not formally examine the subjective experience of the participants, mean accuracy levels were measured at a d′ of 2.42 (maximum possible d′ score was 5.33), which suggests that performance improvement was theoretically possible, supporting the use of our paradigm. Moreover, although our participants appeared to reach a ceiling in performance following practice, applying TES enabled a further improvement, consistent with the literature in which performance in the 2-back task improved in response to TES (Zaehle et al., 2011; Jausovec et al., 2014; Meiron and Lavidor, 2014; Violante et al., 2017). Although greater effects with a higher memory load cannot be excluded, we nonetheless observed a significantly greater improvement in performance after TACS than TDCS. Of additional note is that greater performance enhancement by TDCS was seen on a spatial WM task with greater memory load (Wu et al., 2014), while TACS led to improved performance on the 2-back test but not on the 3-back test (Jausovec et al., 2014). Future studies could address this issue by varying the WM load.

The subgroup analysis is an important feature in our study. As far as we are aware, this study is the first in which a direct comparison was made between the impact of TACS and TDCS on WM performance. Accordingly, we compared the effects of two methods that are both expected to enhance WM performance. In order to detect the differential effects of TACS and TDCS, it was necessary to evaluate performance after participants had already made the substantial improvements in performance due to practice alone. Importantly, pre-stimulation performance in sessions 2 and 3 did not differ significantly according to stimulation type (Figure 4).

We note that the improvement in accuracy over time was variable across participants, and the groups were counterbalanced for gender, age, and IQ. We speculate that the difference is likely to be due to the different strategies developed by different individuals. In the current study, some participants reported a strategy in which they repeated the sequence subvocally, while other participants used a more visually based strategy. With reference to the phonological loop proposed in the Baddeley WM component model, a word or a letter is maintained when a grapheme is translated to a phoneme (Baddeley, 2003). Future work could include a post-study questionnaire regarding strategy development.

Several potential explanations could account for the small WM improvement observed here compared with that reported elsewhere. First is our young, healthy participant group. Previous studies have suggested that participants with lower WM capacity, due to advanced age or cerebral pathology, tend to show greater performance improvement with stimulation (Tseng et al., 2012; Hsu et al., 2014). We chose a young, healthy participant group, however, because our aim was to compare the methods directly, without confounding factors. Our cohort can be seen as “high performing” based on the mean IQ of 120.8, potentially explaining the small improvement with stimulation, although we note that performance did not reach a ceiling effect, meaning our analysis could nonetheless be performed. We also note that the current density using TACS applied here was smaller than that used in the study by Polanía et al. (2012), in which electrode size was 5 cm × 5 cm and current density was 20 μA/cm^2^. Although it is well-known that current density positively correlates with cognitive improvement (Iyer et al., 2005; Nitsche et al., 2008), we chose to employ 7 cm × 5 cm electrodes rather than 5 cm × 5 cm electrodes for two reasons. Firstly, 7 cm × 5 cm is the most commonly used electrode size in the literature (Rostami et al., 2013), and our intention was to apply standard parameters. Second, given that stimulation was applied three times to each participant, the lower current density reduced the probability of side-effects due to skin heating (Nitsche and Paulus, 2000; Nitsche et al., 2008).

Our aim was to match the study parameters for application of TACS and TDCS as closely as possible given the constraint of the fundamental differences between the approaches. The experimental paradigms were identical, and the participants were well-matched for the between-subject comparisons. Matching the current intensity was not possible, however. We had the choice either to apply 1 mA in total for both stimulation types, entailing splitting the 1 mA to 0.5 mA to F3 and 0.5 mA to P3 for TACS, or to apply 1 mA to F3 and 1 mA to P3. The latter would mean doubling the total current intensity applied in the TACS condition. We chose to deliver the same current density in both stimulation conditions, which meant splitting 1 mA across two electrode sites for TACS. It is therefore particularly noteworthy that the greater impact on WM performance was achieved using TACS.

The effects of TES could potentially be increased. The stimulation could be made more focal. Approaches that have been proposed include using a larger return (cathode) electrode (Nitsche et al., 2008), or high definition stimulation could be used (Dmochowski et al., 2011). We also note that individual variation has been observed in the precise frequency of brain oscillations in individuals (Klimesch, 1999; Sweeney-Reed and Nasuto, 2009), and establishing the optimal frequency for TACS for each participant has the potential to provide stronger enhancement of WM performance.

A possible approach to overcoming the issue of the marked effect of practice on task performance, irrespective of the stimulation type used, would be to employ a different WM task for off-line WM performance assessment to the one employed on-line (Andrews et al., 2011). This approach would carry the assumption, however, that the effect of stimulation is a global effect on WM performance, rather than being task-specific, such that a negative effect would have been difficult to interpret.

Alternative study designs could be applied. For example, on-line stimulation effects between groups could be compared, in a between-subject design, with no prior exposure to the 2-back task, including no pre-stimulation task performance. This approach would have resulted in a steep learning curve in all groups, however, rendering such an investigation more suited to an evaluation of learning processes as participants became more proficient in the task than an assessment of WM capacity.

Future studies could also investigate the impact of TACS using different stimulation frequencies. Existing evidence supports the notion that the optimal stimulation frequency is task-specific, with theta-TACS improving WM (Polanía et al., 2012; Jausovec et al., 2014; Meiron and Lavidor, 2014; Violante et al., 2017), beta-TACS increasing short-term memory capacity (Feurra et al., 2016) and also voluntary risky decision making (Yaple et al., 2017), and gamma-TACS enhancing fluid intelligence (Santarnecchi et al., 2013). A recent study comparing theta- and gamma-TACS during an n-back task detected no change in performance following gamma-TACS but a change, albeit inconsistent, following theta-TACS (Pahor and Jausovec, 2018). Gamma (>30 Hz) activity has been associated with local information processing (Jensen et al., 2007), and coupling between theta oscillations and gamma activity has been observed in multiple brain regions during memory processing (Canolty et al., 2006; Axmacher et al., 2010; Sweeney-Reed et al., 2014). TACS applied at low and high frequencies has recently been shown to have a differential effect on cross-frequency coupling (Helfrich et al., 2016), and we postulate that application of TACS to modify the relative timing of theta oscillations and gamma activity could potentially modulate WM performance.

We identified a greater improvement in WM performance following application of theta TACS than anodal TDCS in a within-subject crossover design study, in which the marked effect of practice of the task was eliminated. Our findings support further investigation of TACS to improve WM performance. Because TACS provides the possibility of directly manipulating known oscillatory neural correlates of WM processing, namely frontal-parietal synchrony in the theta frequency range (Sarnthein et al., 1998; Polanía et al., 2012; Sweeney-Reed et al., 2012, 2014), it enables a hypothesis-driven approach to enhancing WM performance. Application of a higher current density, more focal stimulation with other electrode constellations, and employment of individualized stimulation frequencies have the potential of increasing the effect size.

## Author Contributions

CS-R conceived the study. FR, CB, KK, and CS-R designed the study. FR acquired the data. FR and CS-R analyzed and interpreted the data. CB, KR, and KK interpreted the data. FR and CS-R drafted and critically revised the manuscript. CB, KR, KK, HH, and H-JH critically revised the manuscript. All authors read and approved the final manuscript.

## Conflict of Interest Statement

The authors declare that the research was conducted in the absence of any commercial or financial relationships that could be construed as a potential conflict of interest.

## References

[B1] Andrews S. C. Hoy K. E. Enticott P. G. Daskalakis Z. J. Fitzgerald P. B. (2011). Improving working memory: the effect of combining cognitive activity and anodal transcranial direct current stimulation to the left dorsolateral prefrontal cortex. *Brain Stimul.* 4 84–89. 10.1016/j.brs.2010.06.004 21511208

[B2] Antal A. Herrmann C. S. (2016). Transcranial alternating current and random noise stimulation: possible mechanisms. *Neural Plast.* 2016:3616807 10.1155/2016/3616807 27242932 PMC4868897

[B3] Axmacher N. Henseler M. M. Jensen O. Weinreich I. Elger C. E. Fell J. (2010). Cross-frequency coupling supports multi-item working memory in the human hippocampus. *Proc. Natl. Acad. Sci. U.S.A.* 107 3228–3233. 10.1073/pnas.0911531107 20133762 PMC2840289

[B4] Baddeley A. (2003). Working memory: looking back and looking forward. *Nat. Rev. Neurosci.* 4 829–839. 10.1038/nrn1201 14523382

[B5] Baddeley A. Hitch G. (1974). “Working memory,” in *The Psychology of Learning and Motivation: Advances in Research and Theory* ed. Bower G. (New York, NY: Academic Press) 47–89.

[B6] Bindman L. J. Lippold O. C. Redfearn J. W. (1964). The action of brief polarizing currents on the cerebral cortex of the rat (1) during current flow and (2) in the production of long-lasting after-effects. *J. Physiol.* 172 369–382. 10.1234/12345678 14199369 PMC1368854

[B7] Breitling C. Zaehle T. Dannhauer M. Bonath B. Tegelbeckers J. Flechtner H.-H. (2016). Improving interference control in ADHD patients with transcranial direct current stimulation (tDCS). *Front. Cell. Neurosci.* 10:72. 10.3389/fncel.2016.00072 27147964 PMC4834583

[B8] Brunoni A. R. Amadera J. Berbel B. Volz M. S. Rizzerio B. G. Fregni F. (2011). A systematic review on reporting and assessment of adverse effects associated with transcranial direct current stimulation. *Int. J. Neuropsychopharmacol.* 14 1133–1145. 10.1017/S1461145710001690 21320389

[B9] Brunoni A. R. Vanderhasselt M.-A. (2014). Working memory improvement with non-invasive brain stimulation of the dorsolateral prefrontal cortex: a systematic review and meta-analysis. *Brain Cogn.* 86 1–9. 10.1016/j.bandc.2014.01.008 24514153

[B10] Bruyer R. Brysbaert M. (2011). Combining speed and accuracy in cognitive psychology: is the inverse efficiency score (IES) a better dependent variable than the mean reaction time (RT) and the percentage of error (PE)? *Psychol. Belg.* 51 5–13. 10.5334/pb-51-1-5

[B11] Canolty R. T. Edwards E. Dalal S. S. Soltani M. Nagarajan S. S. Kirsch M. S. (2006). High gamma power is phase-locked to theta oscillations in human neocortex. *Science* 313 1626–1628. 10.1126/science.1128115.High 16973878 PMC2628289

[B12] Coffman B. A. Clark V. P. Parasuraman R. (2014). Battery powered thought: enhancement of attention, learning, and memory in healthy adults using transcranial direct current stimulation. *Neuroimage* 85 895–908. 10.1016/j.neuroimage.2013.07.083 23933040

[B13] Creutzfeldt O. D. Fromm G. H. Kapp H. (1962). Influence of transcortical d-c currents on cortical neuronal activity. *Exp. Neurol.* 5 436–452. 10.1016/0014-4886(62)90056-0 13882165

[B14] Dagan M. Herman T. Harrison R. Zhou J. Giladi N. Ruffini G. (2018). Multitarget transcranial direct current stimulation for freezing of gait in Parkinson’s disease. *Mov. Disord.* 33 642–646. 10.1016/j.cogdev.2010.08.003.Personal 29436740 PMC5964604

[B15] Dmochowski J. P. Datta A. Bikson M. Su Y. Parra L. C. (2011). Optimized multi-electrode stimulation increases focality and intensity at target. *J. Neural Eng.* 8:046011. 10.1088/1741-2560/8/4/046011 21659696

[B16] Dundas J. E. Thickbroom G. W. Mastaglia F. L. (2007). Perception of comfort during transcranial DC stimulation: effect of NaCl solution concentration applied to sponge electrodes. *Clin. Neurophysiol.* 118 1166–1170. 10.1016/j.clinph.2007.01.010 17329167

[B17] Feurra M. Galli G. Pavone E. F. Rossi A. Rossi S. (2016). Frequency-specific insight into short-term memory capacity. *J. Neurophysiol.* 116 153–158. 10.1152/jn.01080.2015 27121583 PMC4961742

[B18] Fregni F. Boggio P. S. Nitsche M. Bermpohl F. Antal A. Feredoes E. (2005). Anodal transcranial direct current stimulation of prefrontal cortex enhances working memory. *Exp. Brain Res.* 166 23–30. 10.1007/s00221-005-2334-6 15999258

[B19] Haatveit B. C. Sundet K. Hugdahl K. Ueland T. Melle I. Andreassen O. A. (2010). The validity of d prime as a working memory index: results from the “Bergen n-back task”. *J. Clin. Exp. Neuropsychol.* 32 871–880. 10.1080/13803391003596421 20383801

[B20] Helfrich R. F. Herrmann C. S. Engel A. K. Schneider T. R. (2016). Different coupling modes mediate cortical cross-frequency interactions. *Neuroimage* 140 76–82. 10.1016/j.neuroimage.2015.11.035 26608244

[B21] Herrmann C. S. Rach S. Neuling T. Strüber D. (2013). Transcranial alternating current stimulation: a review of the underlying mechanisms and modulation of cognitive processes. *Front. Hum. Neurosci.* 7:279. 10.3389/fnhum.2013.00279 23785325 PMC3682121

[B22] Hill A. T. Fitzgerald P. B. Hoy K. E. (2016). Effects of anodal transcranial direct current stimulation on working memory: a systematic review and meta-analysis of findings from healthy and neuropsychiatric populations. *Brain Stimul.* 9 197–208. 10.1016/j.brs.2015.10.006 26597929

[B23] Hsu T. Y. Tseng P. Liang W. K. Cheng S. K. Juan C. H. (2014). Transcranial direct current stimulation over right posterior parietal cortex changes prestimulus alpha oscillation in visual short-term memory task. *Neuroimage* 98 306–313. 10.1016/j.neuroimage.2014.04.069 24807400

[B24] Isella V. Molteni F. Mapelli C. Ferrarese C. (2015). Short term memory for single surface features and bindings in ageing: a replication study. *Brain Cogn.* 96 38–42. 10.1016/j.bandc.2015.02.002 25898281

[B25] Iyer M. B. Mattu U. Grafman J. Lomarev M. Sato S. Wassermann E. M. (2005). Safety and cognitive effect of frontal DC brain polarization in healthy individuals. *Neurology* 64 872–875. 10.1212/01.WNL.0000152986.07469.E9 15753425

[B26] Jausovec N. Jausovec K. (2014). Increasing working memory capacity with theta transcranial alternating current stimulation (tACS). *Biol. Psychol.* 96 42–47. 10.1016/j.biopsycho.2013.11.006 24291565

[B27] Jausovec N. Jausovec K. Pahor A. (2014). The influence of theta transcranial alternating current stimulation (tACS) on working memory storage and processing functions. *Acta Psychol.* 146 1–6. 10.1016/j.actpsy.2013.11.011 24361739

[B28] Jensen O. Kaiser J. Lachaux J.-P. (2007). Human gamma-frequency oscillations associated with attention and memory. *Trends Neurosci.* 30 317–324. 10.1016/j.tins.2007.05.001 17499860

[B29] Kim J. H. Kim D. W. Chang W. H. Kim Y. H. Kim K. Im C. H. (2014). Inconsistent outcomes of transcranial direct current stimulation may originate from anatomical differences among individuals: electric field simulation using individual MRI data. *Neurosci. Lett.* 564 6–10. 10.1016/j.neulet.2014.01.054 24508704

[B30] Klimesch W. (1999). EEG alpha and theta oscillations reflect cognitive and memory performance: a review and analysis. *Brain Res. Rev.* 29 169–195. 10.1016/S0165-0173(98)00056-3 10209231

[B31] Lehrl S. Gallwitz A. Blaha V. Fischer B. (1992). *Theorie und Messung der geistigen Leistungsfähigkeit Mit dem Kurztest KAI. 3. Auflage*. Ebersberg: Reihe Psychometrie.

[B32] López-Alonso V. Cheeran B. Río-Rodríguez D. Fernández-Del-Olmo M. (2014). Inter-individual variability in response to non-invasive brain stimulation paradigms. *Brain Stimul.* 7 372–380. 10.1016/j.brs.2014.02.004 24630849

[B33] Mancuso L. E. Ilieva I. P. Hamilton R. H. Farah M. J. (2016). Does transcranial direct current stimulation improve healthy working memory?: A meta-analytic review. *J. Cogn. Neurosci.* 28 1063–1089. 10.1162/jocn 27054400

[B34] Meiron O. Lavidor M. (2014). Prefrontal oscillatory stimulation modulates access to cognitive control references in retrospective metacognitive commentary. *Clin. Neurophysiol.* 125 77–82. 10.1016/j.clinph.2013.06.013 23831184

[B35] Nitsche M. A. Cohen L. Wassermann E. M. Priori A. Lang N. Antal A. (2008). Transcranial direct current stimulation: state of the art 2008. *Brain Stimul.* 1 206–223. 10.1016/j.brs.2008.06.004 20633386

[B36] Nitsche M. A. Paulus W. (2000). Excitability changes induced in the human motor cortex by weak transcranial direct current stimulation. *J. Physiol.* 527 633–639. 10.1111/j.1469-7793.2000.t01-1-00633.x 10990547 PMC2270099

[B37] Ohn S. H. Park C. I. Yoo W. K. Ko M. H. Choi K. P. Kim G. M. (2008). Time-dependent effect of transcranial direct current stimulation on the enhancement of working memory. *Neuroreport* 19 43–47. 10.1097/WNR.0b013e3282f2adfd 18281890

[B38] Owen A. M. McMillan K. M. Laird A. R. Bullmore E. (2005). N-back working memory paradigm: a meta-analysis of normative functional neuroimaging studies. *Hum. Brain Mapp.* 25 46–59. 10.1002/hbm.20131 15846822 PMC6871745

[B39] Pahor A. Jausovec N. (2018). The effects of theta and gamma tACS on working memory and electrophysiology. *Front. Hum. Neurosci.* 11:651. 10.3389/fnhum.2017.00651 29375347 PMC5767723

[B40] Palva J. M. Monto S. Kulashekhar S. Palva S. (2010). Neuronal synchrony reveals working memory networks and predicts individual memory capacity. *Proc. Natl. Acad. Sci. U.S.A.* 107 7580–7585. 10.1073/pnas.0913113107 20368447 PMC2867688

[B41] Polanía R. Nitsche M. A. Korman C. Batsikadze G. Paulus W. (2012). The importance of timing in segregated theta phase-coupling for cognitive performance. *Curr. Biol.* 22 1314–1318. 10.1016/j.cub.2012.05.021 22683259

[B42] Polanía R. Nitsche M. A. Ruff C. C. (2018). Studying and modifying brain function with non-invasive brain stimulation. *Nat. Neurosci.* 21 174–187. 10.1038/s41593-017-0054-4 29311747

[B43] Prabhakaran V. Narayanan K. Zhao Z. Gabriel J. D. E. (2000). Integration of diverse information in working memory within the frontal lobe. *Nat. Neurosci.* 3 85–90. 10.1038/71156 10607400

[B44] Prehn-Kristensen A. Munz M. Göder R. Wilhelm I. Korr K. Vahl W. (2014). Transcranial oscillatory direct current stimulation during sleep improves declarative memory consolidation in children with attention-deficit/hyperactivity disorder to a level comparable to healthy controls. *Brain Stimul.* 7 793–799. 10.1016/j.brs.2014.07.036 25153776

[B45] Rostami M. Golesorkhi M. Ekhtiari H. (2013). Methodological dimensions of transcranial brain stimulation with the electrical current in human. *Basic Clin. Neurosci.* 4 190–208. 25337348 PMC4202570

[B46] Santarnecchi E. Polizzotto N. R. Godone M. Giovannelli F. Feurra M. Matzen L. (2013). Frequency-dependent enhancement of fluid intelligence induced by transcranial oscillatory potentials. *Curr. Biol.* 23 1449–1453. 10.1016/j.cub.2013.06.022 23891115

[B47] Sarnthein J. Petsche H. Rappelsberger P. Shaw G. L. von Stein A. (1998). Synchronization between prefrontal and posterior association cortex during human working memory. *Proc. Natl. Acad. Sci. U.S.A.* 95 7092–7096. 10.1073/pnas.95.12.7092 9618544 PMC22750

[B48] Stagg C. J. Nitsche M. A. (2011). Physiological basis of transcranial direct current stimulation. *Neuroscientist* 17 37–53. 10.1177/1073858410386614 21343407

[B49] Sweeney-Reed C. Zaehle T. Voges J. Schmitt F. Buentjen L. Kopitzki K. (2014). Corticothalamic phase synchrony and cross-frequency coupling predict human memory formation. *eLife* 3:e05352 10.7554/eLife.05352 25535839 PMC4302268

[B50] Sweeney-Reed C. M. Nasuto S. J. (2009). Detection of neural correlates of self-paced motor activity using empirical mode decomposition phase locking analysis. *J. Neurosci. Methods* 184 54–70. 10.1016/j.jneumeth.2009.07.023 19643135

[B51] Sweeney-Reed C. M. Riddell P. M. Ellis J. A. Freeman J. E. Nasuto S. J. (2012). Neural correlates of true and false memory in mild cognitive impairment. *PLoS One* 7:e48357. 10.1371/journal.pone.0048357 23118992 PMC3485202

[B52] Tortella G. Casati R. Aparicio L. V. M. Mantovani A. Senço N. D’Urso G. (2015). Transcranial direct current stimulation in psychiatric disorders. *World J. Psychiatry* 5 88–102. 10.5498/wjp.v5.i1.88 25815258 PMC4369553

[B53] Townsend J. Ashby F. (1978). “Methods of modeling capacity in simple processing systems,” in *Cognitive Theory* Vol. 3 eds Castellan J. Restle F. (Hillsdale, N.J: Erlbaum) 200–239.

[B54] Tseng P. Hsu T.-Y. Chang C.-F. Tzeng O. J. L. Hung D. L. Muggleton N. G. (2012). Unleashing potential: transcranial direct current stimulation over the right posterior parietal cortex improves change detection in low-performing individuals. *J. Neurosci.* 32 10554–10561. 10.1523/JNEUROSCI.0362-12.2012 22855805 PMC6621415

[B55] Violante I. R. Li L. M. Carmichael D. W. Lorenz R. Leech R. Hampshire A. (2017). Externally induced frontoparietal synchronization modulates network dynamics and enhances working memory performance. *eLife* 6:e22001. 10.7554/eLife.22001 28288700 PMC5349849

[B56] Wiethoff S. Hamada M. Rothwell J. C. (2014). Variability in response to transcranial direct current stimulation of the motor cortex. *Brain Stimul.* 7 468–475. 10.1016/j.brs.2014.02.003 24630848

[B57] Wu Y. J. Tseng P. Chang C. F. Pai M. C. Hsu K. Sen Lin C. C. (2014). Modulating the interference effect on spatial working memory by applying transcranial direct current stimulation over the right dorsolateral prefrontal cortex. *Brain Cogn.* 91 87–94. 10.1016/j.bandc.2014.09.002 25265321

[B58] Yaple Z. Martinez-Saito M. Feurra M. Shestakova A. Klucharev V. (2017). Transcranial alternating current stimulation modulates risky decision making in a frequency-controlled experiment. *Eneuro* 4:ENEURO.136-ENEURO.117. 10.1523/ENEURO.0136-17.2017 29379865 PMC5779115

[B59] Zaehle T. Sandmann P. Thorne J. D. Jäncke L. Herrmann C. S. (2011). Transcranial direct current stimulation of the prefrontal cortex modulates working memory performance: combined behavioural and electrophysiological evidence. *BMC Neurosci.* 12:2. 10.1186/1471-2202-12-2 21211016 PMC3024225

